# 3D-NASE: A Novel 3D CT Nasal Attention-Based Segmentation Ensemble

**DOI:** 10.3390/jimaging11050148

**Published:** 2025-05-07

**Authors:** Alessandro Pani, Luca Zedda, Davide Antonio Mura, Andrea Loddo, Cecilia Di Ruberto

**Affiliations:** Department of Mathematics and Computer Science, University of Cagliari, Via Ospedale 72, 09124 Cagliari, Italy; davideantonio.mura@unica.it (D.A.M.); cecilia.dir@unica.it (C.D.R.)

**Keywords:** 3D CT segmentation, nasal CT, 3D U-Net, UNETR, Swin UNETR, ensemble methods

## Abstract

Accurate segmentation of the nasal cavity and paranasal sinuses in CT scans is crucial for disease assessment, treatment planning, and surgical navigation. It also facilitates the advanced computational modeling of airflow dynamics and enhances endoscopic surgery preparation. This work presents a novel ensemble framework for 3D nasal CT segmentation that synergistically combines CNN-based and transformer-based architectures, 3D-NASE. By integrating 3D U-Net, UNETR, Swin UNETR, SegResNet, DAF3D, and V-Net with majority and soft voting strategies, our approach leverages both local details and global context to improve segmentation accuracy and robustness. Results on the NasalSeg dataset demonstrate that the proposed ensemble method surpasses previous state-of-the-art results by achieving a 35.88% improvement in the DICE score and reducing the standard deviation by 4.53%. These promising results highlight the potential of our method to advance clinical workflows in diagnosis, treatment planning, and surgical navigation while also promoting further research into computationally efficient and highly accurate segmentation techniques.

## 1. Introduction

An accurate understanding of nasal cavity and paranasal sinus morphology is vital for clinical applications, disease detection, treatment planning, endonasal surgery simulations, or surgical navigation. For instance, the precise delineation of regions such as the nasal cavity and paranasal sinuses in computed tomography (CT) scans is vital for assessing sinus-related diseases [1], planning endoscopic surgeries [2], and enabling advanced computational models of airflow dynamics [3,4].

Segmentation data are key in diagnosis, surgical preparation, and robotic system navigation. Volume and surface area quantification help assess nasal health and the impact of inhaled substances [5]. Also, detailed anatomical knowledge supports endoscopic sinus surgery, guiding structural reconstruction and outcome evaluation. Although manual segmentation has long been considered the gold standard in clinical practice, it is inherently labor-intensive and prone to both inter- and intra-observer variability. These limitations have fueled the search for robust, automated segmentation approaches that can deliver consistent and reproducible results. In this context, a precise, automated segmentation of anatomical structures in 3D medical imaging becomes critical for effective diagnosis, treatment planning, and surgical navigation [5,6].

Furthermore, the recent surge in radiomics research has underscored the importance of high-quality segmentation as a foundation for extracting reliable quantitative imaging features [7]. Radiomics seeks to convert medical images into high-dimensional data by extracting features that capture subtle tissue heterogeneity and underlying pathophysiological processes. The spectrum of features ranges from classical handcrafted descriptors, such as shape, first-order intensity statistics, and texture measures, to sophisticated representations learned via deep neural networks, including convolutional neural networks (CNNs), recurrent neural networks (RNNs), and transformer-based architectures. Such diversity in feature representation plays a crucial role in enhancing diagnostic accuracy, prognostication, and the prediction of treatment responses.

Moreover, the introduction of novel datasets, such as the NasalSeg [8] dataset, has begun to address the scarcity of large-scale, annotated imaging data necessary for training and benchmarking advanced segmentation models. This work proposes an ensemble-based segmentation framework that synergistically combines multiple 3D architectures, 3D-NASE. Our approach integrates CNN-based models with transformer-based networks that exploit self-attention mechanisms. By fusing these complementary architectures, our method aims to enhance segmentation robustness and improve generalization across anatomical variations and heterogeneous imaging conditions. It aims not only to refine segmentation accuracy but also to provide a reliable basis for subsequent radiomic analysis. In summary, the research question we aim to address in this work is *how can an ensemble-based segmentation framework that integrates both CNN-based and transformer-based architectures be designed to enhance the accuracy, robustness, and reproducibility of 3D nasal CT segmentation for improved clinical diagnosis and treatment planning?*

The remainder of the paper is organized as follows. In Section 2, we review the state-of-the-art radiomic segmentation by discussing both 2D and 3D approaches and the emerging trends and challenges in the field. Section 3 details the materials and methods used in our study, including the architectures of U-Net, 3D U-Net, UNETR, and Swin UNETR, along with the description of the NasalSeg dataset and the evaluation metrics. In Section 4, we introduce 3D-NASE and outline the experimental setup. Section 5 presents the quantitative experimental results, while Section 6 offers a qualitative analysis of the segmentation outputs. We discuss the limitations of our approach in Section 7 and conclude the paper with final remarks and directions for future work in Section 8.

## 2. Related Work

Over the past decade, significant progress has been made in radiomic segmentation through contributions from traditional image processing, machine learning, and deep learning methodologies. This section provides a comprehensive review of the state-of-the-art segmentation techniques in radiomics. The discussion is organized into four subsections, with a focus on the 2D (Section 2.1) and 3D (Section 2.2) segmentation approaches, along with a discussion of their distinctions and complementarities (Section 2.3), and future developments (Section 2.4).

### 2.1. Two-Dimensional Radiomic Segmentation

Two-dimensional segmentation techniques have been extensively explored due to their simplicity and relatively low computational burden. Early methods predominantly relied on classical image processing techniques such as thresholding, region growing, edge detection, and active contour models [9,10]. These methods proved effective when target structures exhibited high contrast and well-defined boundaries.

More recently, the emergence of deep learning models has shifted the common approaches toward more automatic and fast architectures capable of segmenting clinically relevant regions in different imaging techniques [11,12].

However, despite their computational efficiency and ease of implementation, 2D segmentation methods inherently lack the ability to fully exploit the volumetric context present in modern imaging modalities [13]. This shortcoming limits their utility in applications for which three-dimensional structural continuity [14] is critical, such as in tumor delineation or the assessment of complex anatomical regions.

### 2.2. Three-Dimensional Radiomic Segmentation

High-resolution volumetric imaging has spurred a paradigm shift toward 3D segmentation methods. Unlike 2D approaches, 3D segmentation takes advantage of the complete spatial context, resulting in a more comprehensive and accurate delineation of anatomical structures [13,15]. Volumetric methods are particularly indispensable in modalities like CT and magnetic resonance (MR) imaging, where the intricate spatial relationships between tissues must be preserved.

Deep learning has become the cornerstone of modern 3D segmentation [16,17]. The 3D U-Net architecture [18], for example, has emerged as a benchmark due to its encoder–decoder structure, which facilitates the capture of both local and global contexts across multiple scales. In addition to CNN-based models, transformer-based architectures have recently been introduced to model long-range dependencies across the volume [19]. With their self-attention mechanisms, these models are adept at identifying subtle variations and heterogeneities within the data, which are often missed when conventional convolutional filters are used [20].

Hybrid approaches have also been proposed, combining the rapid processing of 2D methods with the spatial accuracy of 3D segmentation [21,22]. Typically, these frameworks commence with a 2D segmentation step to quickly identify candidate regions, followed by a refined 3D segmentation to ensure volumetric consistency. This two-tiered strategy effectively balances the trade-offs between computational efficiency and segmentation accuracy [23], making it highly attractive for real-time clinical applications [24].

### 2.3. Comparative Analysis and Challenges

A comparative analysis between 2D and 3D segmentation techniques reveals a range of trade-offs. While 2D methods offer simplicity and speed [25,26], they are limited by their inability to capture inter-slice contextual information. On the other hand, 3D approaches, despite their higher computational demands, provide a more faithful representation of anatomical structures [27,28]. One of the primary challenges with 3D segmentation is the substantial computational overhead associated with processing volumetric data. High-resolution images require large amounts of memory and extended training times, often necessitating specialized hardware or cloud-based resources.

Another significant challenge is the variability in imaging protocols and acquisition parameters across different clinical centers. This variability can lead to discrepancies in image quality and intensity distributions, thereby affecting the generalizability of segmentation models [29]. To address these issues, recent research has focused on developing standardized preprocessing pipelines and data harmonization techniques essential for mitigating inter-scanner and inter-center variations.

Furthermore, the integration of segmentation outputs into radiomic pipelines poses additional challenges. High-quality segmentation is a prerequisite for reliable feature extraction, as errors in delineation can propagate and degrade the performance of downstream predictive models [30]. As such, the development of explainable and robust segmentation methods is a critical area of ongoing research.

### 2.4. Emerging Trends and Future Directions

The field of radiomic segmentation is rapidly evolving, driven by innovations that bridge the gap between traditional methods and modern deep learning techniques. One emerging trend is the integration of self-supervised learning and foundation models [31,32], which aim to leverage large-scale unannotated data to improve model robustness and reduce the reliance on extensively labeled datasets. These approaches are particularly promising for addressing the limitations posed by data scarcity in medical imaging [33,34].

Another exciting direction is the development of multi-modal segmentation frameworks. By fusing complementary information from different imaging modalities such as CT, MR, and positron emission tomography (PET), researchers are beginning to overcome the limitations inherent in single-modality analysis. Multi-modal fusion techniques can enhance the accuracy and reliability of segmentation, particularly in complex clinical scenarios where different modalities provide unique and complementary insights [35,36].

## 3. Materials and Methods

In this section, we present the materials and methods employed in our study, focusing on the architectures used for medical image segmentation (Section 3.1, Section 3.2, Section 3.3 and Section 3.4). We detail these networks’ theoretical foundations and practical adaptations to handle volumetric data, which is essential for accurately delineating complex anatomical structures. In addition, we provide a dataset description in Section 3.9 and the metrics definition in Section 3.10.

### 3.1. U-Net

U-Net [37] is a popular biomedical image segmentation architecture with an encoder–decoder structure. In the encoding stage, the network progressively reduces the spatial dimensions of the input through a series of convolutional layers followed by a downsampling operation. For instance, if $f^{l}$ denotes the feature map at layer *l*, a typical downsampling can be expressed as

$$f^{l+1}=D\left(f^{l}\right),$$

where *D* might represent a max pooling operation (e.g., with a stride of two) or a convolution with a stride greater than one, the selected U-Net architecture uses strided convolution as the downsampling operator. This reduction not only decreases computational complexity but also helps in capturing contextual information over larger regions. In the decoding stage, the spatial resolution is gradually recovered using an upsampling operation. Mathematically, if $g^{l+1}$ is the feature map at a deeper layer in the decoder, then upsampling can be described as

$$g^{l}=U\left(g^{l+1}\right),$$

where *U* may be implemented via transposed convolutions or interpolation, followed by convolution. The network further refines the segmentation by incorporating skip connections that merge the high-resolution features from the encoder with the upsampled features in the decoder, thereby preserving fine structural details.

### 3.2. Three-Dimensional U-Net

Three-Dimensional U-Net [18] extends the principles of U-Net to volumetric data by replacing two-dimensional operations with their three-dimensional counterparts. In this architecture, the downsampling operator is adapted to process volumetric feature maps. If $f^{l}$ is the 3D feature map at layer *l*, the volumetric downsampling is represented as

$$f^{l+1}=D_{3D}\left(f^{l}\right),$$

where $D_{3D}$ aggregates information along the depth, height, and width dimensions simultaneously. Similarly, the upsampling operator in the decoder is defined as

$$g^{l}=U_{3D}\left(g^{l+1}\right),$$

which reconstructs the spatial dimensions of the volume, again using transposed convolutions or interpolation methods adapted to three dimensions. The integration of skip connections in 3D U-Net allows the network to effectively combine coarse, context-rich features with finer details, thereby enabling the accurate segmentation of complex anatomical structures in modalities such as CT and MRI.

### 3.3. UNETR

UNETR [19] integrates a transformer-based [38] encoder into the U-Net framework to capture long-range dependencies in volumetric data. The input volume *x* is first divided into non-overlapping patches, each of which is embedded into a token via a linear projection, i.e.,

$$t_{i}=\phi \left(x_{i}\right),$$

where $x_{i}$ is the *i*th patch, and ϕ denotes the embedding function. The transformer encoder then processes the sequence of tokens using self-attention, which is computed as

$$\mathrm{Attention}\left(Q,K,V\right)=\mathrm{softmax}\left(\frac{QK^{⊤}}{\sqrt{d_{k}}}\right)V,$$

with *Q*, *K*, and *V* being the query, key, and value matrices, respectively, and $d_{k}$ the key dimension. The resulting encoded representation is reshaped and fed into a U-Net style decoder that recovers spatial resolution through an upsampling operation

$$g^{l}=U\left(g^{l+1}\right),$$

while incorporating skip connections to merge high-resolution features from the encoder. This design enables UNETR to effectively combine global context with local details. In our work, we follow the original UNETR implementation [19]. Specifically, feature maps are extracted from five distinct layers of the transformer encoder. These multi-scale features are fused in the upsampling branch by first applying deconvolution to match the spatial dimensions of both the backbone and the target feature maps. This is subsequently refined through a sequence of convolutional operations, batch normalization, and ReLU activations, which collectively improve the training process.

### 3.4. Swin UNETR

Swin UNETR [20] extends the UNETR framework by employing the Swin Transformer [39] architecture, which utilizes a hierarchical structure with window-based self-attention. In this model, the input volume is partitioned into patches, and self-attention is computed locally within non-overlapping windows. The attention mechanism is the same as defined in [19] applied independently over each window. A subsequent shift in the window partitioning enables the capture of cross-window dependencies, ensuring that global context is effectively modeled. Following the transformer encoding, a decoder analogous to that of UNETR performs upsampling. This approach synergizes local attention with global context, thereby improving the segmentation of complex volumetric data [20].

### 3.5. SegResNet

SegResNet [40] is a modification of the traditional ResNet architecture tailored to semantic segmentation tasks. This architecture incorporates residual connections, which help mitigate the vanishing gradient problem by allowing gradients to flow through the network without degradation. The encoding path consists of a series of extensive residual blocks that learn multiscale features from the input images, progressively reducing the spatial dimensions. The unique aspect of SegResNet is its autoencoder portion of the architecture, which improves the final segmentation prediction according to [40]. In our implementation, we only rely on the segmentation loss and not on the reconstruction.

### 3.6. DAF3D

DAF3D [41] introduces a novel approach for robust 3D medical image segmentation by utilizing deep attentive features within a convolutional neural network framework. The architecture leverages attention mechanisms to automatically focus on relevant parts of the input volumes, enhancing segmentation performance by prioritizing critical features while suppressing less informative ones. The network operates through a series of convolutional layers that capture intricate patterns in the volumetric data, supported by a dedicated attention module that refines feature representations based on channel-wise and spatial relationships.

### 3.7. V-Net

V-Net [42] employs a fully convolutional neural network architecture specifically designed for volumetric segmentation tasks. The structure follows a similar encoder–decoder paradigm while utilizing 3D convolutions to process volumetric data directly. In the encoder portion, V-Net incorporates residual learning blocks that facilitate the training of deeper networks by enhancing gradient flow. The decoder then upsamples the feature maps via transposed convolution and combines them with high-resolution features from the encoder via skip connections, allowing for precise localization.

### 3.8. Advantages and Disadvantages of the Proposed Networks

Our study utilizes six different 3D segmentation architectures: 3D U-Net, UNETR, Swin UNETR, SegResNet, DAF3D, and V-Net. Among these, the 3D U-Net, V-Net, SegResNet, and DAF3D are CNN-based models that rely on convolutional operations. These models are renowned for their robust ability to extract fine-grained local features and are computationally efficient. However, their dependence on convolutions also results in a limited receptive field, which can restrict the capture of long-range contextual information. This limitation may lead to challenges such as over-segmentation, particularly in complex anatomical regions.

In contrast, UNETR incorporates transformer-based techniques that excel at modeling global contexts. By leveraging self-attention mechanisms, UNETR can capture long-range dependencies that are crucial for understanding subtle variations and complex spatial relationships in volumetric data. Although this approach improves the representation of broader contextual information, it comes at the cost of increased computational complexity and demands a larger training dataset. The process of tokenizing volumetric data adds further preprocessing intricacies, which can be challenging compared to traditional CNNs [43].

Swin UNETR builds upon the strengths of both convolutional and transformer architectures by incorporating a hierarchical structure with window-based self-attention. This design allows the model to effectively capture local patterns within non-overlapping windows, while also integrating global context through a shifting mechanism. Swin UNETR thus achieves a balance between detailed feature extraction and comprehensive contextual integration. However, this balance introduces additional model complexity and requires careful tuning of window sizes and shift parameters to optimize performance [43].

The ensemble framework we propose—3D-NASE—combines these six networks to take advantage of their complementary benefits, also following consolidated literature works that previously combined the characteristics of both model typologies [44]. By integrating the local feature precision and global feature from both CNN and transformer-based models, our ensemble is designed to counteract the individual limitations of each architecture. The combination, implemented via majority and soft voting strategies, ensures improved segmentation accuracy and robustness across diverse anatomical variations and imaging conditions, ultimately enhancing the overall performance of the segmentation task.

### 3.9. Dataset

The NasalSeg dataset [8] is a large-scale, open-access resource developed for the automatic segmentation of the nasal cavity and paranasal sinuses from 3D CT images. It comprises 130 head CT scans collected from independent patients, including 74 males and 56 females, with an age range of 24 to 82 years (mean 54.6±12.1 years). All scans were acquired using a Biograph 64 scanner (Siemens, Erlangen, Germany) at the Department of Nuclear Medicine/PET Center, Huashan Hospital, and they typically exhibit a volume of 148×512×512 pixels, with an in-plane resolution of 0.586×0.586 mm and a slice spacing of 1.5 mm.

Each scan is accompanied by pixel-wise annotations of five anatomical structures: the left nasal cavity, right nasal cavity, nasal pharynx, left maxillary sinus, and right maxillary sinus. These annotations were initially performed by three skilled annotators with one to five years of experience and subsequently refined and verified by senior experts, with five to ten years of experience, to ensure high accuracy and consistency. The manual annotation and refined segmentation procedure was adopted from 3D Slicer 5.6.2 (3D Slicer: www.slicer.org/, accessed on 6 March 2025). The air threshold was set to −400 to 1000 Hounsfield units (HU) for all scans. The Laplacian filter was then used to highlight regions with rapid intensity changes to enhance edges and facilitate the segmentation. The annotations were verified by four experienced experts specializing in radiology and otolaryngology (with over 20 years of experience).

Furthermore, the dataset has been released as organized into five pre-defined folds to support cross-validation and robust benchmarking of segmentation algorithms. Each set consists of 104 cases for training and 26 cases for validation.

**Figure 1:**
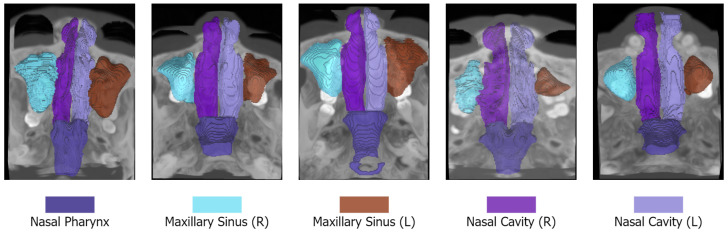
Samples from the NasalSeg dataset along with corresponding labels.

We report samples of the NasalSeg dataset along with over-imposed annotations in Figure 1.

### 3.10. Metrics

We evaluate our 3D segmentation results using several quantitative metrics that capture different aspects of performance. For a binary segmentation task, we define the following basic terms: TP represents true positives (the number of correctly identified positive elements), TN represents true negatives (the correctly identified negative elements), FP represents false positives (negative elements incorrectly labeled as positive), and FN represents false negatives (positive elements that were missed). In multi-class segmentation, these metrics are computed for each class individually and then averaged to yield an overall performance score without weighting the number of items in each class. This strategy ensures that performance is fairly assessed even when class distributions vary, and, in the context of this work, this decision was made to ensure that each anatomical structure is treated equally during evaluation.

The quality of the segmentation overlap is measured by the DICE coefficient, which is calculated as

$$\mathrm{DICE}=\frac{2TP}{2TP+FP+FN}$$

This metric is widely adopted in medical image segmentation because it effectively captures the degree of overlap between the predicted segmentation and the ground truth. A DICE score of 1 indicates perfect overlap, while scores closer to 0 reflect poor agreement. The formulation penalizes both FPs and FNs equally, making it a robust indicator of segmentation quality.

Another critical measure is the intersection over union (IoU), which, for a single class, is defined as

$$\mathrm{IoU}=\frac{TP}{TP+FP+FN}$$

The IoU quantifies the ratio of the intersection between the predicted and actual segmentation regions relative to their union, offering an intuitive measure of error when extra regions are included or parts of the target are missed. For multi-class segmentation, the mean intersection over union (mIoU) is calculated by averaging the IoU values across all classes:

$$\mathrm{mIoU}=\frac{1}{C}\sum_{c=1}^{C}\frac{TP_{c}}{TP_{c}+FP_{c}+FN_{c}}$$

where *C* is the total number of classes, and the subscript *c* indicates that the metric is computed for each class separately. This aggregate metric is particularly useful in evaluating the overall performance across diverse anatomical structures.

To further analyze segmentation performance, we employ sensitivity (or recall) to assess the proportion of actual positive elements that are correctly identified:

$$\mathrm{Sensitivity}=\frac{TP}{TP+FN}$$

A high sensitivity is crucial, especially in medical applications, because it ensures that most of the true positives are captured, thereby reducing the risk of missing critical regions.

Complementary to sensitivity, specificity measures the proportion of actual negative elements that are correctly identified:

$$\mathrm{Specificity}=\frac{TN}{TN+FP}$$

High specificity is important for minimizing the inclusion of irrelevant regions in the segmentation, which is particularly critical when false positives can lead to misinterpretations.

Precision, also known as positive predictive value (PPV), evaluates the accuracy of the positive predictions:

$$\mathrm{PPV}=\frac{TP}{TP+FP}$$

This metric indicates the proportion of predicted positive elements that are indeed correct. High precision reflects the reliability of the segmentation in not overestimating the presence of the target structure.

Similarly, the negative predictive value (NPV) assesses the correctness of the negative predictions:

$$\mathrm{NPV}=\frac{TN}{TN+FN}$$

A high NPV ensures that the segmentation method reliably identifies non-target areas, which is essential for maintaining overall segmentation integrity.

Finally, the overall performance of the segmentation is summarized by the accuracy metric:

$$\mathrm{Accuracy}=\frac{TP+TN}{TP+TN+FP+FN}$$

Accuracy measures the proportion of correctly classified elements (both positive and negative) out of all elements. Although it provides a global performance view, it should be interpreted alongside the other metrics, especially in scenarios with class imbalance.

Together, these metrics offer a comprehensive framework for assessing segmentation performance. They not only quantify the spatial overlap between the predicted and ground truth segmentations but also provide insights into the balance between correctly identifying positive elements and avoiding erroneous classifications. This multi-faceted evaluation is critical for refining segmentation algorithms, particularly in high-stakes applications such as medical imaging.

## 4. The Proposed Framework: 3D-NASE

**Figure 2:**
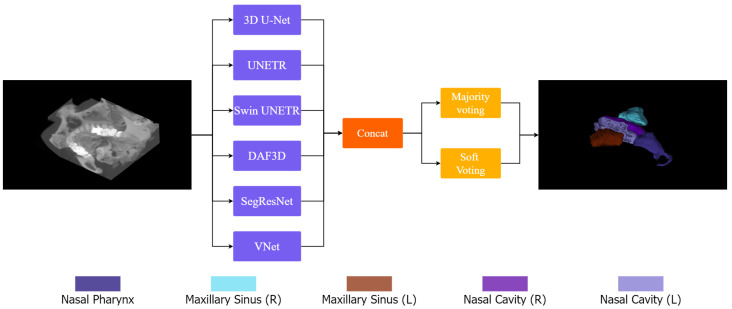
The proposed framework, 3D-NASE: the input volume is processed in parallel by the selected models, their outputs are concatenated, and an ensemble strategy is applied to generate the final prediction.

Ensemble learning has emerged as a promising strategy for 3D segmentation in medical imaging due to its ability to reduce model variance, improve generalization, and alleviate artifacts commonly encountered in high-dimensional volumetric data. In our proposed framework, 3D-NASE, we harness the complementary strengths of both CNN-based and transformer-based architectures by integrating six state-of-the-art segmentation models: 3D U-Net, UNETR, Swin UNETR, SegResNet, DAF3D, and V-Net. Each of these models is specifically designed to capture distinct aspects of the data—while the CNN-based networks 3D U-Net, SegResNet, DAF3D, and V-Net excel at extracting fine-grained local details, the transformer-based models UNETR and Swin UNETR are adept at modeling long-range dependencies and global contextual information via self-attention mechanisms. By processing the same CT volume in parallel, these models generate segmentation outputs that are subsequently aggregated using both majority and soft voting strategies. This dual ensembling approach leverages diverse feature representations across different architectures, effectively balancing their individual strengths and limitations. Figure 2 schematically illustrates our pipeline, where the input volume is processed by the selected models, their outputs concatenated, and an ensemble strategy is applied to generate the final prediction.

**Experimental Setup.** We conducted our experiments on a workstation equipped with an RTX 4060 Ti GPU, 16GB VRAM, and an Intel Core i5-13400 processor. All models were trained for 800 epochs with a learning rate of $3\times 10^{-4}$, and the best models were selected based on the highest DICE score on the validation set. 3D-NASE was built upon the MONAI repository [45]. For optimization, we employed AdamW, together with the DICE loss function.

Data augmentation was an integral part of our training process. Specifically, we utilized two common strategies: random cropping and intensity normalization. For random cropping, a random volume crop with a region of interest (ROI) of 96×96×32 voxels was extracted from each input image. After an analysis performed over the size of the images, it was identified that an ROI of 96×96×32 allows a significant portion of the anatomical structure of interest to be captured while reducing the variability introduced by irrelevant or background regions. Intensity normalization was performed on a per-channel basis by considering only non-zero voxels, scaling their intensities to have a mean of 0 and a standard deviation of 1. We do not consider other augmentation strategies in order to focus our results analysis on the impact of the ensemble techniques for a more stable prediction framework, rather than artificially improving upon the quantity of the data towards the same goal.

## 5. Experimental Results

This section presents the experimental evaluation conducted and the results obtained. We evaluated the employed architectures as stand-alone and the proposed ensemble-based framework across different folds since every experiment uses a five-fold cross-validation approach to ensure a fair assessment and mitigate potential biases. The complete set of experiments across folds is presented in Table 1, where we also compared the results with the baseline provided in [8]. As can be seen, the proposed soft voting approach improves the DICE score by 35.88% with respect to the baseline (94.43% against 58.55%) and reduces the standard deviation by 4.52% (from 5.43% of the baseline to 0.91%), thereby highlighting enhanced adaptability and stability under varying training conditions. Moreover, the ensemble strategy achieves a 0.37% improvement over Swin UNETR, the best stand-alone model. Although the improvement is marginal, these results illustrate that leveraging the diverse knowledge acquired by different models leads to more reliable outcomes.

Notably, the current state of the art for the NasalSeg dataset [8] exhibits lower performance than individual models and ensemble strategies regarding the DICE score and demonstrates higher variability across folds.

Extending the evaluation to a broader range of segmentation metrics, Table 2 demonstrates that 3D-NASE precisely segments the relevant regions, as indicated by high accuracy, specificity, and NPV; however, it tends to under-segment the analyzed volumes, except for the nasal pharynx, which suffers from over-segmentation. These insights are further supported by the results provided in Table 3, which shows the DICE score for the five different classes.

Among the classes with similar performance, the right and left nasal cavities are the most challenging to segment, likely due to intra-observer differences observed in the model predictions. This aspect is evidenced by the average DICE score and the higher standard deviation for these two classes, which is an order of magnitude greater than that for the other classes.

## 6. Qualitative Results

**Figure 3:**
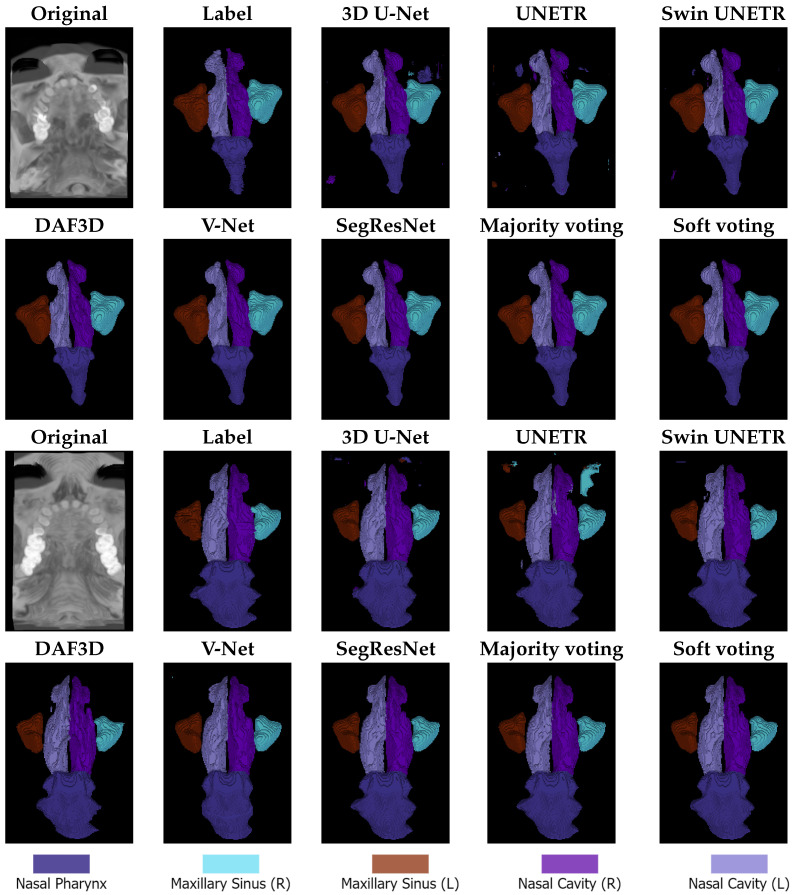
Qualitative results: original images, ground-truth labels, and predictions from 3D U-Net, UNETR, Swin UNETR, DAF3D, V-Net, SegResNet, majority voting, and soft voting. The first two rows represent the first example, while the last two rows represent the second one.

The qualitative results are presented in Figure 3. All models exhibit a tendency toward over-segmentation for the nasal pharynx. This issue is particularly evident in 3D U-Net and UNETR, which also often predict small, scattered label fragments across different image regions. These fragmented predictions result in artifacts and inconsistencies in the final segmentation masks.

The proposed ensemble methods effectively mitigate this issue by filtering out small-scale imperfections, leading to more refined and coherent segmentation. However, larger over-segmentation artifacts tend to persist even in the ensemble predictions, indicating that, while ensemble strategies improve overall segmentation quality, they may not entirely eliminate over-segmentation when it occurs on a larger scale.

The qualitative results presented in Figure 3 align with the observations made during the discussion of the quantitative results. Specifically, the regions most affected by segmentation errors are the right and left nasal cavities. These structures are either misclassified as other anatomical classes, as seen in the U-Net prediction for the second image, or other classes are mistakenly predicted as nasal cavities, as observed in the U-Net prediction for the third image.

These findings further reinforce the quantitative evaluation, highlighting the challenges associated with segmenting the nasal cavity regions. This suggests that their complex shape, small size, and similarity in intensity to surrounding structures contribute to the models’ difficulty in accurately distinguishing them.

## 7. Limitations and Future Aspects

While our proposed framework for nasal segmentation demonstrates significant improvements over the current state of the art, it also presents some limitations.

The first limitation of our approach is the modest improvement provided by the ensemble techniques over the Swin UNETR architecture. While the soft voting ensemble achieves a DICE score of 94.43%, an incremental gain of 0.54 compared to Swin UNETR—a difference that is not statistically significant, but, as shown in Figure 3, the ensemble notably enhances the prediction quality by substantially reducing artifacts.

It still exhibits artifacts and instances of over-segmentation and under-segmentation that vary across different anatomical classes. Such inconsistencies can compromise the reliability of the segmentation outputs, particularly when delineating complex or subtle structures. To improve these aspects, we plan further investigations. For instance, implementing post-processing steps such as conditional random fields (CRFs) or connected-component analysis can be helpful in removing scattered label fragments and refining the borders of segmented regions. Additionally, experimenting with loss functions like Tversky loss may better balance false positives and negatives during the training phase.

Another critical limitation is the necessity for a large number of training epochs to achieve satisfactory results. This high computational demand can hinder accessibility, especially for smaller institutions or research groups with limited resources in a fine-tuning scenario. The extensive training requirements not only prolong the development cycle but also increase the overall cost of deployment. To take this issue into account, we plan to explore techniques such as progressive learning rate scheduling and early stopping to reduce training duration without compromising performance.

Moreover, while the ensemble methodology effectively combines CNN- and transformer-based architectures, it requires all six selected models to be trained on the same dataset and perform inference on the same volume. This integrated approach further compounds computational and memory resource requirements, posing a significant challenge in scenarios where real-time performance is essential. Moreover, the marginal performance improvement warrants further exploration. For instance, we intend to assess alternative ensembling methods, such as stacked generalization or weighted voting schemes, for which model predictions are weighted based on performance per anatomical region. This could lead to a more balanced segmentation performance across different regions.

Finally, since CT images can vary due to differences in acquisition protocols or patient positioning and the absence of further public datasets dealing with this task, we will simulate varying imaging conditions during training and testing. Introducing synthetic variations (e.g., changes in resolution or noise levels) will assess the model’s robustness under different scenarios.

Future work should focus on optimizing training strategies to reduce the number of required epochs, as well as exploring more resource-efficient ensemble methods. By addressing these issues, it may be possible to maintain or even enhance segmentation performance while reducing the computational burden, thus broadening the applicability of the framework in resource-constrained environments.

## 8. Conclusions

In this work, we have introduced 3D-NASE, an ensemble-based segmentation framework that integrates the complementary strengths of CNN and attention-based architectures for 3D nasal CT segmentation. By merging predictions from 3D U-Net, UNETR, Swin UNETR, SegResNet, DAF3D, and V-Net through majority and soft voting, our method effectively captures both fine-grained details and long-range contextual information. Experimental results on the NasalSeg dataset reveal that our ensemble approach not only outperforms individual models but also improves the overall state-of-the-art segmentation performance, with a notable 35.88% increase in the DICE score and a 4.52% reduction in variability across cross-validation folds. Despite these significant gains, challenges such as over-segmentation artifacts and high computational demands persist. Future work will focus on optimizing the training pipeline and exploring more resource-efficient ensemble strategies to further enhance segmentation accuracy and efficiency, thus broadening the clinical applicability of the proposed framework.

## Figures and Tables

**Table 1:** DICE scores across folds demonstrate that 3D-NASE with soft voting approach outperforms the current state-of-the-art on the NasalSeg dataset by 35.88%. Notably, ensemble methods achieve superior segmentation performance compared to individual networks. The best results are emphasized in bold.

Fold#	0↑	1↑	2↑	3↑	4↑	AVG↑
Baseline [8]	56.25	65.70	62.31	58.73	49.76	58.55 ± 5.43
3D U-Net	94.21	93.22	90.93	92.61	94.75	93.15 ± 1.49
UNETR	93.51	92.48	92.49	92.01	94.07	92.92 ± 0.85
Swin UNETR	94.65	93.57	93.58	92.98	94.67	93.89 ± 0.74
DAF3D	92.47	91.59	91.45	91.03	93.11	91.93 ± 0.84
SegResNet	94.42	93.23	91.21	92.47	94.62	93.19 ± 1.41
VNet	92.95	92.19	89.56	91.67	93.48	91.98 ± 1.51
3D-NASE (Majority voting)	95.17	93.92	**94.07**	93.41	95.47	94.41 ± 0.88
3D-NASE (Soft voting)	**95.21**	**93.94**	93.95	**93.46**	**95.58**	**94.43 ± 0.91**

**Table 2:** Quantitative comparison of segmentation performance across different models on the NasalSeg dataset. The soft voting ensemble achieves the highest DICE, mIoU, and Sensitivity, outperforming individual networks. Additionally, ensemble methods maintain high specificity and accuracy, demonstrating their effectiveness in producing reliable segmentation results. The best results are emphasized in bold.

Method	DICE↑	mIoU↑	Sensitivity↑	Specificity↑	Accuracy↑	PPV↑	NPV↑
3D U-Net	93.15 ± 1.49	88.57 ± 1.61	92.75 ± 2.02	98.82 ± 0.36	99.64 ± 0.04	93.95 ± 0.81	99.03 ± 0.14
UNETR	92.92 ± 0.85	87.89 ± 0.86	93.61 ± 0.88	98.97 ± 0.08	99.62 ± 0.03	92.51 ± 0.95	98.81 ± 0.14
Swin UNETR	93.89 ± 0.74	89.91 ± 0.59	94.54 ± 0.90	98.92 ± 0.04	99.12 ± 0.02	93.58 ± 0.64	98.65 ± 0.15
DAF3D	91.93 ± 0.84	86.32 ± 0.84	92.79 ± 0.93	98.76 ± 0.17	99.54 ± 0.02	91.29 ± 0.87	98.50 ± 0.06
SegResNet	93.19 ± 1.41	88.59 ± 1.57	94.06 ± 1.69	99.05 ± 0.23	99.63 ± 0.05	92.57 ± 1.24	98.78 ± 0.28
VNet	91.98 ± 1.51	86.63 ± 1.64	92.25 ± 1.59	98.68 ± 0.35	99.57 ± 0.05	92.05 ± 1.37	98.66 ± 0.28
3D-NASE (majority voting)	94.41 ± 0.88	90.43 ± 0.83	**95.10 ± 1.04**	**99.23 ± 0.11**	**99.70 ± 0.02**	93.94 ± 0.69	99.02 ± 0.09
3D-NASE (soft voting)	**94.43 ± 0.91**	**90.46 ± 0.94**	**95.10 ± 1.08**	99.14 ± 0.14	**99.70 ± 0.02**	**94.51 ± 0.72**	**99.13 ± 0.08**

**Table 3:** DICE scores for each anatomical class segmented by different models on the NasalSeg dataset. The soft voting ensemble achieves the highest DICE scores across all classes, demonstrating superior segmentation performance for the maxillary sinuses, nasal cavities, and nasal pharynx. The best results are emphasized in bold.

Method	Background↑	Maxillary Sinus (R)↑	Maxillary Sinus (L)↑	Nasal Cavity (R)↑	Nasal Cavity (L)↑	Nasal Pharynx↑
3D U-Net	99.48 ± 0.69	94.91 ± 1.72	94.21 ± 2.68	88.33 ± 3.06	88.20 ± 2.77	93.77 ± 2.15
UNETR	99.44 ± 0.41	93.78 ± 0.83	94.13 ± 1.97	88.42 ± 2.93	88.07 ± 2.89	93.65 ± 1.06
Swin UNETR	99.59 ± 0.43	94.71 ± 1.05	94.28 ± 1.89	89.89 ± 2.75	89.82 ± 2.78	95.03 ± 1.06
DAF3D	99.30 ± 0.38	95.32 ± 0.67	94.52 ± 1.71	83.91 ± 2.76	83.66 ± 2.70	94.90 ± 0.91
SegResNet	99.46 ± 0.86	94.81 ± 2.08	94.08 ± 2.75	88.40 ± 3.07	88.34 ± 3.07	94.07 ± 1.57
VNet	99.35 ± 0.95	94.02 ± 2.81	93.14 ± 2.91	86.19 ± 2.91	86.06 ± 2.67	93.09 ± 2.18
3D-NASE (Majority voting)	**99.56 ± 0.87**	95.93 ± 2.08	**95.10 ± 2.91**	90.23 ± 2.64	**90.17 ± 2.78**	95.51 ± 1.18
3D-NASE (Soft voting)	**99.56 ± 0.91**	**95.94 ± 2.97**	95.09 ± 2.86	**90.28 ± 2.83**	**90.17 ± 2.77**	**95.54 ± 1.83**

## Data Availability

All the material used and developed for this work is available at the following GitHub repository (https://github.com/snarci/YOLO-SPAM, accessed on 6 March 2025).

## Abbreviations

The following abbreviations are used in this manuscript:

CT	Computed Tomography
MR	Magnetic Resonance
PET	Positron Emission Tomography
CNN	Convolutional Neural Network
RNN	Recurrent Neural Network
TP	True Positive
FP	False Positive
FN	False Negative
IoU	Intersection over Union
PPV	Positive Predictive Value
NPV	Negative Predictive Value
ROI	Region of Interest
